# A report on the housing vervet monkeys adjacent to domestic cats as a means of environmental enrichment

**DOI:** 10.4102/ojvr.v87i1.1870

**Published:** 2020-11-26

**Authors:** John K. Chipangura, Andre Ganswindt, Vinny Naidoo

**Affiliations:** 1 Biomedical Research Centre, Faculty of Veterinary Science, University of Pretoria, Pretoria, South Africa; 2 Department of Paraclinical Sciences, Faculty of Veterinary Science, University of Pretoria, Pretoria, South Africa; 3 Department of Anatomy and Physiology, Faculty of Veterinary Science, University of Pretoria, Pretoria, South Africa; 4 Mammal Research Institute, Faculty of Natural and Agricultural Sciences, University of Pretoria, Pretoria, South Africa

**Keywords:** vervet monkeys, domestic cats, stress, faecal glucocorticoid metabolites

## Abstract

In current research guidelines, much focus is placed on ethical management of animals and the application of principles of reduction, refinement and replacement. Of these refinements through environmental enrichment is an important aspect when housing primate to prevent behavioural problems. In this study, we investigated the co-housing of domestic cats and vervet monkeys as a novel method of enrichment based on the cohabitation and stress alleviation effect of horses housed with goats and from seeing cats cohabitating with vervet monkeys in an animal sanctuary. The study used a habituation method whereby the cats were stepwise introduced to the monkeys by sight and smell but with physical separation. Assessment included changes in behaviour, weight and faecal glucocorticoid metabolite (fGCM) concentrations over time. On the first day of housing, the vervets whilst inquisitive kept their distance. The vervets housed in cages that were closest to the cats were the most active and during the first minute of introduction made more alarm calls, which stopped a few days later. The fGCMs were non-significantly different. The results of this study provide evidence that vervet monkeys and domestic cats could potentially be housed together without overt aggression. We thus suggest further observations to ascertain if the co-housing could have long-term benefits for vervet monkeys, from the companionship that would be offered by the cats.

## Introduction

Experimental animals play an important role in biomedical research. Whilst the use of animal models is meant to generate reliable results that could allow for extrapolation to humans or veterinary medicine, stress in the research animals can at times become a confounder. These stressful conditions tend to confound research data by altering the physiological status of the animal (Hubrecht & Kirkwood 2010). For laboratory animals held in captivity, housing and environmental conditions can become the stressors. Stress can impact the well-being of animals by resulting in effects such weight loss, immune system dysregulation and even aggression (Honess & Marin 2006).

To improve these circumstances, good welfare practice advocates for the full implementation of the 3Rs (reduction, replacement, refinement) in research (Hubrecht & Kirkwood 2010; Russell & Burch 1959). Of the 3Rs, refinement is aimed at methods used to minimise the severity of handling of animals in research and thus the stress experienced by animals (Newberry 1995; Olsson & Dahlborn 2002). Examples of refinement techniques include gentle handling and providing animals with appropriate housing that allows for the expression of species-specific behaviours (Newberry 1995; Olsson & Dahlborn 2002), with enrichment of the home environment also being an essential component. Whilst the housing conditions for laboratory animals may be improved through methods such as the provision of toys, logic puzzles and offering different types of bedding, the most important enrichment tool is to provide an environment where animals would have an opportunity to express species-specific behaviours, which for primates is social housing – an important aspect to consider (Coleman & Novak 2017; Hubrecht & Kirkwood 2010). For proper social housing, one requires adequate space that not only takes into account floor space by height because of the arboreal nature of primate natural environments. Unfortunately, the latter is not always possible in research centres, as adequate space is a limiting factor for economic reasons and when taking into account disease mitigation strategies, which would require wholly indoor housing.

Vervet monkeys (*Chlorocebus pygerythrus*) are one common non-human primate (NHP) species used in biomedical research (Jorgensen et al. 2017). Despite their widespread use, there is limited information available describing their optimal environmental enrichment. With vervets in the wild living in large social groups, it is not surprising that these animals’ social needs require attention when in captivity to prevent behavioural abnormalities, whilst at the same time maintaining the integrity and quality of research data (Seelig 2007). In contrast, the rhesus macaque (*Macaca mulatta*) represents the most commonly used NHP species in research and numerous published studies exist on their optimal enrichment in captivity (e.g. Coleman & Novak 2017; Novak et al. 1998; Reinhardt 1999; Weed et al. 2003; Wooddell et al. 2019). As a result, enrichment methods developed for macaques are often adopted for vervet monkeys. Whilst these interventions may be successful, generalisations on husbandry of NHPs can lead to further problems because the animals’ psychological response to interventions may be very species-specific.

The University of Pretoria Biomedical Research Centre (UPBRC) housed adult vervet monkeys over a period of 10 years, for which numerous environmental enrichment techniques have been tried on the colony. The specific facility had five primate rooms of which four were in use. Each room had an indoor and outdoor area. Whilst the indoor area is one large space per primate room, the indoor area can if needed be subdivided into smaller cage spaces. Both the indoor and outdoor areas are sufficiently high for the placement of branches to stimulate arboreal behaviour. Whilst the indoor areas are physically separated by brick walls, the outdoor pens are separated by a double fence system. As a result of the permanent housing of these primates, our laboratory is constantly looking into ways to better enrich the environment of these permanently housed primates. A potential opportunity presented itself when a university graduate student wanted to undertake research on disease-free cats. In a laboratory animal research environment, the optimal housing conditions for cats should allow for sufficient space, allow for group housing and provide for opportunities for behaviours such as climbing (Geret et al. 2011). Based on the similarity in housing requirements between cats and NHPs, we asked the question as to whether the animals could be housed in the same environment and whether both species would benefit from their interaction. We based this supposition on a previous study that showed the cohabitation and stress alleviation effect of housing horses with goats (Winter 1996). In our consideration, we felt that the cats would be minimally intimidating to the NHPs because of their size.

We also undertook an extensive literature review, where we found no information on co-housing of cats and primates. At present the only information we were able to find on interspecies co-housing was for different NHP species in the same cage, which were for rhesus and cynomolgus macaques (Rehrig & Wyatt 2012). As a result, a general Internet search was undertaken, where numerous reports of primates and cats forming strong bonds could be found, together with one specific mention of a vervet baby bonding with two cats at a sanctuary (Carlson 2017). Furthermore, we had the opportunity to visit a local vervet sanctuary in South Africa, where we were able to observe a group of feral cats making their home in the enclosure with no adverse interactions between the two groups. No information was also available on the potential for disease transmission between the two species. For this study, we describe our experience with the housing and the results from stress-monitoring testing that was also undertaken, as a pilot study to look at the potential of co-housing of a primate and non-primate species.

## Materials and methods

### Animals used in the study

#### Vervet monkeys

**FIGURE 1:**
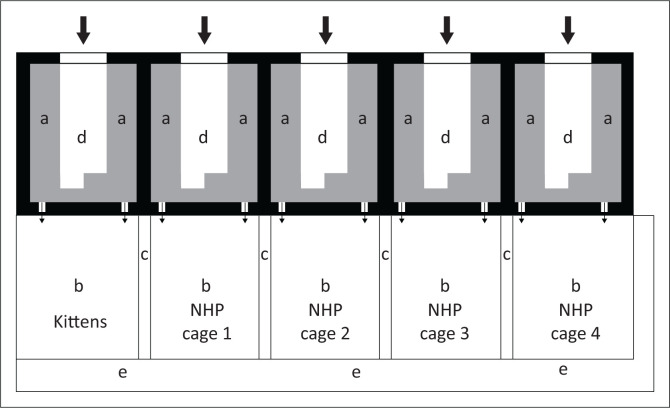
Cage layout for the cats and vervet monkeys. (a) inside cage area; (b) outside pens; (c) double fence separating the outer pens; (d) inside observation area; (e) outside observation area; thick arrow, room entry point; thin arrow, animal outer access hatches; large solid areas, bricked walls; thin line, expanded metal mesh.

The vervets used in this study were resident at the UPBRC and were not part of any other research study at the time of the experiment. The colony is unique in that the individual history of the animals is unknown, including their age and relationship with each other, as the animals were all wild caught at an unknown date before being moved to their current home. The animals were resident at the UPBRC since 2003 and were estimated to have an age of 9 years when they arrived at the facility. The study animals were thus likely very close to their lifespan of typically 25 years in captivity. The colony comprised six females and four males. At the time of their arrival, the animals were housed in individual commercial primate cages. Since then their habitat has been modified to a design that includes both indoor and outdoor areas (Figure 1). The colony is currently kept in heterosexual pairs or groups of three in ceiling height (3 m height) cages and surface area of 5.7 m^2^, with an indoor and outdoor component since 2011, with both enclosures having artificial trees therein to the current period of 2017 when the study was undertaken. The vervets had free choice of selecting being indoors or outdoors at any time of the day. The cages were cleaned at least once a day during the observational period and the vervets fed twice daily, in the morning with fruits and primate biscuits (wheat bran, maize meal, protein, vitamin, mineral (PVM) primate supplement (PVM Nutritional Sciences, South Africa) and vitamin C (Junglevites Chewey C, PharmaNatura, South Africa) and in the afternoon with fruits and vegetables. Potable municipal water was provided *ad libitum*. All male vervets were castrated in 2011 prior to heterosexual pair housing. Room temperature for the indoor cages was maintained at 20 ºC – 25 ºC, with a relative air humidity of approximately 50% and a 12-h artificial light/dark cycle. Food enrichment was provided in the form of raisins, rusks, sunflower seeds and nuts and environmental enrichment by providing hard plastic toys, balls, foraging containers, plastic crates, climbing wooden logs, puzzle feeders, swing ropes and tyres.

#### Domestic cats

Eight neutered domestic cats that were assigned to form part of another long-term study, which has been postponed, were used. The cats were 6 weeks old at the start of housing, and were not exposed to monkeys before. The colony comprised four females and four males. Before the experiment, the cats were group housed in the same unit as the primate, albeit in a separate room (room 5). The room as for the primates was equipped with an indoor and outdoor section, water bowl, food bowl, litter tray, cat beds and soft blankets. The enclosure was cleaned at least once a day. The cats were fed twice a day with a commercial cat diet and water was provided *ad libitum*. Environmental enrichment included scratch poles, tree stumps, hiding places and commercial cat toys. The inner room was separated from the primates by brick wall, whilst for safety reasons the outdoor areas of the primates and cats were separated by a movable double expanded metal grids, with a 30 cm separation between the two grid layers.

### Experimental procedures

Six days prior to housing the animal species next to each other separated by a barrier, the vervets and cats were monitored in their accustomed enclosures with behavioural observations and faecal sample collection taking place three times per day. Further animals were weighed a day before the housing experiment began to establish baseline weights. The vervets were well accustomed to being weighed as this was a common procedure during their housing at the facility and they participated in the procedure without any aggression or overt signs of stress. For the weighing the animals entered into a smaller cage of known weight on wheels, which is subsequently weighed on a calibrated platform scale. The vervet monkeys were kept in their usual social groups, during the introduction of the domestic cats as next-door neighbours. No changes were made to the animals’ diets and water was provided *ad libitum*. The cats were allowed outdoor access to the enclosure next to the vervets during the day (8:00–14:00), whilst they were returned to their indoor housing at night for the first month. After 1 month, the cats were left permanently in the enclosure next to the vervets, with a free choice of inside or outside access. To prevent injuries, the cats and vervets were never in direct contact, with a double expanded-metal mesh separating them always, but they could see and smell each other via the cage fencing. To minimise confounding variables, only personnel whom the primates were accustomed to could work in the primate and cat unit. Staff members, responsible for monitoring the animals, had at least 3 years of experience working with the vervets and were the same staff who took care of the kittens. During the first week of introduction, we monitored the domestic cat and vervet behaviour on an hourly basis, which we decreased to twice daily during the second week to ensure that the animals did not injure themselves. To prevent bias in behavioural observations, behaviours of the animals were monitored by closed-circuit video.

### Behavioural observations

The cats and vervets were monitored for the full duration of the experiment by closed-circuit video (Hikvision DVR – Model:DS-9016HFI-S [Hikvision corporate solutions, Alberton, South Africa] and Samsung camera – Model: SHC-721AP [Samsung Electronics, Johannesburg, South Africa]). As we had no idea about what behaviour the animals would elicit on the introduction, we decided to monitor them against predator avoidance behaviour, which was previously described. It was believed that if the vervets were to be stressed by the presence of the cats, they would elicit a typical predator avoidance behaviour. We also made the assumption that the vervet monkeys may associate the domestic cats with servals (*Felis serval*), which are their natural predators, although the two species are different in size (Guy & Curnoe 2013).The following behaviours were chosen for analysis, as described by Seyfarth, Cheney and Marler (1980) as relevant for primates facing a potential predator as looking in direction of predator: alarm calls defined as short tonal calls produced in a series of inhalations and exhalations or climbing high up tree branches provided in the enclosure. For alarm calls the total number of calls was recorded, whilst for the other behaviours the number of animals showing the behaviour was recorded.

### Faecal sample collection

Faecal samples from both species were collected for 6 days prior to co-housing to determine species-specific baseline values. After introducing the domestic cats, faeces were collected for both species for another 6 days. The enclosures were inspected three times a day, in the morning (7:00–10:00), at noon and afternoon (14:00–15:00) and all faeces were collected into individually labelled plastic bottles. Samples were homogenised using a wooden spatula before placing into plastic bottles. All available samples, including samples produced overnight, were collected. All samples were stored at −20 °C within 1 h of collection until analysis.

For the vervets, individual sampling was possible because individuals were fed biscuits mixed with different food colorants (Robertsons, Durban, South Africa). The domestic cats used communal litter trays and the faecal sample could only be identified to a particular cat when the cat was observed defecating. In total 311 samples were collected for the vervets and 79 for the cats.

### Faecal steroid extraction and analysis

At the Endocrine Research Laboratory, Faculty of Veterinary Science, University of Pretoria, South Africa, the frozen faecal samples were lyophilised, pulverised and sifted through a wire-mesh strainer to separate faecal powder from undigested material. About 0.10 g – 0.11 g faecal powder from each sample was then extracted by adding 3 mL 80% (volume/volume) ethanol. The suspension was vortexed for 15 min and subsequently centrifuged at 1500 × *g* for 10 min. The supernatant (1.5 mL) was then transferred into labelled Eppendorf safe-lock micro test tubes and stored at -20 °C until further analysis.

Faecal steroid extracts were measured for faecal glucocorticoid metabolite (fGCMs) concentrations, using established enzyme immunoassays (EIA) for fGCM monitoring in cats (Schatz & Palme 2001) and vervet monkeys (Young et al. 2017). Respective EIAs used antibodies against 11-oxoetiocholanolone (detecting 11, 17-dioxoandrostanes, for the cats) and cortisol (for the vervet monkeys). Detailed assay characteristics, including a full description of the assay components and cross-reactivities, are provided for both EIAs by Palme and Möstl (1997). Assay procedures followed the protocols published by Ganswindt et al. (2002). Sensitivity of the 11-oxoetiocholanolone EIA was 2.4 ng/g dry weight (DW)and for the cortisol EIA it was 0.6 ng/g DW. Intra-assay coefficients of variation determined by repeated measurements of high and low value quality controls were 4.8% and 5.8% for 11-oxoetiocholanolone and 4.0% and 4.8% for cortisol measurements. Inter-assay coefficients of variation were 8.1% and 12.6% for 11-oxoetiocholanolone and 12.7% and 14.7% for the cortisol EIA.

### Statistical analysis

The behaviour of the vervets was monitored on the closed-circuit video recording and then analysed using descriptive statistics prior to cat introduction, immediately post-introduction, and after 3 days of housing. To check for significant changes in behaviour before and during co-housing, the data were checked for normality using the Shapiro–Wilk test and analysed using the Kruskal–Wallis (KW) test. One-way analysis of variance (ANOVA) on ranks and multiple comparisons using Tukey’s post hoc test were used to isolate the group or groups that differ from others. Differences in animal weight before and after 6 days of co-housing were examined for cats and vervets using either a Wilcoxon signed rank or paired *t*-test. Differences in fGCM concentrations for the day prior to introduction, of introduction and days 1 and 2 post-introduction for cats and vervets were analysed using KW one-way ANOVA on ranks (for the cats as no individual sampling could be conducted) or repeated measured ANOVA (for the vervets). The individual median fGCM concentration was calculated in cases where more than one sample per day was collected from an individual. Normality of available data sets was analysed using Shapiro–Wilk’s test. The statistical analyses were performed using the software programme Sigma Plot 12.5. The significance level was set at 0.05.

### Ethical consideration

The University of Pretoria Animal Ethics Committee (UP-AEC) approved the use of vervet monkeys and domestic cats that were resident at the UPBRC for this study (Protocol Number V118/15). The UP-AEC is registered with the South African National Health Research Ethics Council (NHREC), and follows the South African National Standard for the care of research animals (SANS10386).

## Results

### Behavioural observations

On the first day of housing next to cats, it was observed that the vervets, in general whilst interested with the presence of the cats (looking in direction of cats, pacing up and down), still maintained their distance from the cage boundary. The four vervets housed in two cages that were closest to the domestic cats, with only 30 cm (group 1) and 3 m (group 2) between the enclosures, respectively, were the most active. During the first 1 min of introduction, the vervets looked more often at the cats, climbed up to the top of the cage and made more alarm calls (Table 1). The calls were made whilst looking in the direction of the cats, with the other vervets in the cages furthest from the cats responding by looking at the direction of the caller. The calls were significantly reduced by day 3 and no calls were made by day 14. None of the vervets showed redirected aggression to cage mates and no aggression was evident for any other point of the study. The cats demonstrated inquisitive behaviour as they explored the new environment although without urine marking. We attributed the absence of urine markers rather to the habituation of the cats to using a litter tray.

**TABLE 1:** Frequency of recorded responses of the vervets.

Respondent location	Number of leopard alarm calls recorded*			Number of vervets looking in the direction of cats*			Number of vervets climbing up the tree branches		
	One minute before cats introduced	Cats introduced	Three days after cats introduced	One minute before cats introduced	Cats introduced	Three days after cats introduced	One minute before cats introduced	Cats introduced	Three days after cats introduced
Cage 1	0	43	20	1	2	1	1	2	2
Cage 2	3	37	12	2	2	2	2	2	2
Cage 3	1	25	18	1	3	0	0	3	1
Cage 4	6	12	7	0	3	1	2	3	2

The response with an asterisk (*) had a statistically significant difference after performing the ANOVA test (leopard alarm calls, [*p* = 0.001]; looking in the direction of cats, *p* = 0.029).

When comparing the number of alarm calls made 1 min before the cats were introduced, at the time when the cats were introduced and 3 days after the cats were introduced, the difference in the median values (2, 31 and 15, respectively) amongst the groups was found to be greater than that would be expected by chance (*p* = 0.001), and the pairwise multiple comparison test showed evidence of a statistically significant difference only for 1 min before the cats were introduced and at the time the cats were introduced. For the number of vervets looking in the direction of the cats, the difference in the median values amongst the groups was greater than that would be expected by chance (*p* = 0.029), and the pairwise multiple comparison test showed evidence of a statistically significant difference only for 1 min before the cats were introduced and at the time the cats were introduced.

### Weight alterations related to changes in housing conditions

There was a no significant (*t* = 0.218, *df* = 9, *p* = 0.832) increase in average weight for the vervets from 5.5 kg ± 0.82 kg (mean ± standard deviation [SD]) before housing to 5.6 kg ± 0.81 kg during housing (Table 2). In contrast, there was a significant increase (*W* = 36, *n* = 8, *p* < 0.001) in weight gain for the cats during housing, with the overall average weight of 3.7 kg ± 0.72 kg prior compared with 3.9 kg ± 0.73 kg during co-housing (Table 3). The latter was as expected for young growing cats.

**TABLE 2:** Vervet monkeys’ weight (kg) before and during housing next to domestic cats.

Vervet ID	Sex	Weight before co-housing	Weight during co-housing	Cage
V 1	Male	5.8	6.0	Cage 1
V 2	Female	5.8	5.6	
V 3	Female	6.8	6.6	Cage 2
V 4	Male	5.8	5.8	
V 5	Female	4.9	4.8	Cage 3
V 6	Female	5.0	5.0	
V 7	Male	6.8	6.8	
V 8	Male	5.6	5.4	Cage 4
V 9	Female	4.0	4.2	
V 10	Female	5.1	5.0	

**TABLE 3:** Domestic cats’ weight (kg) before and during housing next to vervet monkeys.

Cat ID	Sex	Weight before co-housing	Weight during co-housing
C 1	Female	3.4	3.6
C 2	Male	4.1	4.3
C 3	Female	4.6	4.8
C 4	Male	4.8	5.0
C 5	Female	3.0	3.2
C 6	Female	3.1	3.2
C 7	Male	3.0	3.2
C 8	Male	3.9	4.1

### Glucocorticoid alterations related to changes in housing conditions

There was no significant (*F* = 0.59, *n* = 10, *p* = 0.627) difference in fGCM levels for the vervets before, during and on days 1 and 2 post-introduction, with the overall mean fGCM concentrations of 95.22 ± 43.04 ng/g DW (mean ± SD) prior and 116.49 ± 62.07 ng/g DW during co-housing. Similarly, there was no significant (*H* = 2.04, *df* = 3, *p* = 0.564) difference in fGCM levels for the cats before, during and on days 1 and 2 post-introduction), with the overall mean fGCM concentrations of 0.33 ± 0.17 *µ*g/g DW (mean ± SD) prior and 0.38 ± 0.28 *µ*g/g DW during co-housing.

## Discussion

This study was aimed at evaluating the potential of housing vervet monkeys (*C. pygerythrus*) next to domestic cats (*Felis silvestris catus*), which we believed would be appropriate based on the similar housing requirements between these species, the size of the cats and an observed natural instance at a local vervet sanctuary where the two species tolerated each other well. Despite the potential benefits of companionship to both the species related to reduction in stress, because of the possibility of the animals responding adversely to the interaction including aggression or self-injury, we ran the experiment as a pilot study, with a fence being maintained between the two groups, and used faecal glucocorticoids as one of the main indicator parameters.

On the first day of housing, both vervets and domestic cats were aware of the other species although they kept their distance. No aggression was evident in any of the cage groupings. The monkeys (two males and two females) housed in the cages that were closest to the domestic cats were the most vocal. Our findings of no aggression are similar to those of Jorgensen et al. (2017), who noted that vervets rarely show overt aggression on the day of introduction to new housing system when pair housed with another vervet. The vervets however responded in a similar way reported for animals in the wild when they encounter leopards (Seyfarth et al. 1980) by showing an increase in directed looking and climbing behaviour and making alarm calls. The alarm calls were clearly directed towards the cats as the caller looked directly at the cats, with the animals in the furthest enclosure looking at the direction of the caller. This was an unexpected finding as the vervets have been in captivity for over 15 years and it indicates that alarm calls were likely a learnt behaviour during infancy to avoid predators (Seyfarth et al. 1980). With the animals becoming accustomed to the cats in what can be considered no longer than a routine acclimatisation period, this would indicate that cats could be companions to primates. However, the next step would be to evaluate the direct interactions of the two species to ascertain if companionship bond is formed as seen in the report of a vervet baby raised with cats.

Another important parameter in the monitoring of animal welfare is their change in body weight, which may increase or decrease. Animals can lose weight (Poole 1997) because of inappetence or a reduction in food and water intake. Even when the animal maintains a normal appetite during stress, the underlying causes of stress may increase energy expenditure, contributing to a net loss of energy that will subsequently lead to weight loss (Hubrecht & Kirkwood 2010; Poole 1997). In contrast, the animals may have an increase in weight because of cortisol-mediated deposition of fat in the abdominal region together with increased liver weights. With none of the animals inducted into the study having lost weight, it can thus be concluded that housing vervet monkeys and domestic cats separated by a physical barrier was not a stressful event to either species.

With three pivotal parameters of stress (weight loss, faecal glucocorticoid concentrations and behavioural parameters) all being relatively stable, the results of this study provide the first step in demonstrating that vervets and domestic cats could be co-housed and this can be used as a way of utilising limited laboratory animal space. However, as the study relied on physical separation, the next step would be to explore the possibilities of co-housing vervets and cats in the same enclosure.
